# Transcriptome Profiling Identifies Differentially Expressed Genes in Skeletal Muscle Development in Native Chinese Ducks

**DOI:** 10.3390/genes15010052

**Published:** 2023-12-28

**Authors:** Yuchen Zhang, Yinglin Lu, Minli Yu, Jin Wang, Xubin Du, Dong Zhao, Huifang Pian, Zongliang He, Guansuo Wu, Shiwei Li, Sike Wang, Debing Yu

**Affiliations:** 1 Department of Animal Genetics, Breeding and Reproduction, College of Animal Science and Technology, Nanjing Agricultural University, Nanjing 210095, China; 2022105039@stu.njau.edu.cn (Y.Z.);; 2 School of Animal Medical, Jiangsu Agri-Animal Husbandry Vocational College, Taizhou 225300, China; 3 Nanjing Academy of Animal Husbandry and Poultry, Nanjing 210095, China; 4 College of Animal Science, Xizang Agricultural and Animal Husbandry University, Linzhi 860000, China

**Keywords:** RNA-seq, skeletal muscle, embryonic period, candidate genes, key signaling pathways

## Abstract

China boasts a rich diversity of indigenous duck species, some of which exhibit desirable economic traits. Here, we generated transcriptome sequencing datasets of breast muscle tissue samples from 1D of four groups: Pekin duck pure breeding group (P), Jinling White duck breeding group (J), P ♂ × J ♀ orthogonal group (PJ) and J ♂ × P ♀ reciprocal-cross group (JP) (*n* = 3), chosen based on the distinctive characteristics of duck muscle development during the embryonic period. We identified 5053 differentially expressed genes (DEGs) among the four groups. Network prediction analysis showed that ribosome and oxidative phosphorylation-related genes were the most enriched, and muscular protein-related genes were found in the 14-day-old embryonic group. We found that previously characterized functional genes, such as *FN1*, *AGRN*, *ADNAMST3*, *APOB* and *FGF9*, were potentially involved in muscle development in 14-day-old embryos. Functional enrichment analysis suggested that genes that participated in molecular function and cell component and key signaling pathways (e.g., hippo, ribosome, oxidative phosphorylation) were significantly enriched in the development of skeletal muscle at 14 days of embryonic age. These results indicate a possible role of muscle metabolism and *myog*lobin synthesis in skeletal muscle development in both duck parents and hybrids.

## 1. Introduction

Skeletal muscle serves as a crucial genetic indicator in the breeding process and holds significant economic value in livestock production. Skeletal muscle in the chordate embryo originates from a transient somite in the mesoderm [1]. Myogenic stem cells within the somite express the myogenic regulatory factors (*MRFs*) and undergo differentiation into myoblasts [2]. Following differentiation, myoblasts continue to fuse, forming multinuclear muscle tubes, which subsequently undergo additional fusion and differentiation to develop into mature muscle fibers [3]. This intricate series of events, encompassing migration, proliferation, differentiation and fusion, collectively constitutes the process known as myogenesis during embryonic development [4]. A series of genes control this process, such as paired Box 3 (*Pax*3), which select migrating myoblast progenitor cells in the transient “skin sarcomere” and run through the whole *myog*enic process in the form of *Pax*7. In addition, the myogenic regulatory factor (*MRF*) family, myocyte enhancer Factor 2 (*MEF2*) family, insulin-like growth factors (*IGFs*), and follistatin (*FST*) are involved in the regulation of myocyte generation and development by orderly expression [5,6,7].

Similar to most animals, poultry skeletal muscle fibers undergo formation during the embryonic stage and do not experience an increase after birth. The growth of skeletal muscle during the growing period primarily relies on the hypertrophy of muscle fibers triggered by the cascade of muscle satellite cells [8]. Therefore, muscle development in avian embryos plays a crucial role in the subsequent muscle growth during the growing period.

However, the mechanism of skeletal muscle development in duck embryos remains obscure. As a nonmodel aquatic fowl, the skeletal muscle development of ducks differs from that of chickens in the embryonic stage. For example, the embryonic period of chickens is only 21 days, whereas the breast muscles of ducks show growth arrest and even atrophy after 21 days (the late stage of embryonic development). The reason might be the lack of continuous nutritional supply in the late stage of embryonic development, which led to a significant decrease in breast muscle mass, muscle fiber cross-sectional area and other indicators from 22 days after embryo development and a significant increase until 7 days after incubation. However, the expression levels of protein degradation-related genes and transcription factors showed opposite results. By comparative analysis of the breast transcriptome sequencing results of E13, E19 and E27 of Pekin duck, it was found that the differentially expressed genes (DEGs) identified in the early stage were significantly fewer than those in the late stage. The DEGs in the early stage were mainly involved in cell metabolism and cell division, while the DEGs in the late stage were mainly enriched in many pathways related to fat development [9]. While the skeletal muscle development processes are similar in Gaoyou ducks and Jind-ing ducks, there are notable differences in their development rates and initial body weights [10]. Therefore, the growth and development of skeletal muscle in duck embryos is the key to studying the difference between breeds. With the continuous development and improvement of second-generation high-throughput sequencing technology, RNA transcriptome sequencing (RNA-seq) technology is a commonly used and comprehensive method to study gene expression levels [11,12]. Through transcriptome sequencing analysis of the differences in breast and leg muscles of 15-day embryos of Pekin duck and black duck, we found that DEGs were only significantly enriched in the ribosomal signaling pathway between breeds [13].

In this study, the offspring of the Pekin duck pure breeding group (P), Jinling White duck breeding group (J), Pekin duck ♂ × Jinling White duck ♀ orthogonal group (PJ) and Jinling White duck ♂ × Pekin duck ♀ reciprocal-cross group (JP) were used as the research subjects. Jinling White ducks are a hybrid breed of Beijing ducks and Liancheng White ducks, combining the high meat quality of Chinese native ducks and the fast growth rate of Beijing ducks. The 14-, 21- and 28-day-old male duck embryos were used to extract RNA from the breast muscle tissue and reverse transcribed into cDNA for high-throughput sequencing. Through transcriptome sequencing analysis, for the first time, we compared the gene expression levels of hybrid duck breast muscle and their parents in the middle and late stages of embryo development, obtained a relatively complete genetic map of the duck transcriptome and screened the key candidate genes and protein interaction networks that caused the differences in skeletal muscle growth and development. Our study laid a foundation for further study on gene expression regulation in hybrid populations and provided a theoretical basis for the hybrid breeding process.

## 2. Materials and Methods

### 2.1. Sample Collection and Ethics Statement

In this experiment, animal welfare was fully considered and carried out in accordance with the requirements of the Ethical Committee and the Institutional Animal Care and Use Committee of Nanjing Agricultural University, Nanjing, China (Certification No.: SYXK(Su)2021-0086). Jinling White ducks (tentative name) were obtained by crossbreeding between purebred Liancheng White ducks (female parent) and Cherry Valley ducks (male duck). All duck eggs of the four breeding groups (P, J, PJ and JP) were collected for five days (incubation temperature: 37.8 °C, humidity: 70%). At 14, 21 and 28 days after hatching, the breast muscles of 3 male duck embryos with similar development status were taken from each group at each stage and then frozen in liquid nitrogen to obtain a total of 36 breast tissue samples. One side of the breast was used for transcriptome sequencing, and the other side was placed in the freezer at −80 °C to facilitate the verification of the follow-up results.

### 2.2. RNA Extraction, Library Construction and Sequencing

RNA sequencing with high quality (8.5 ≤ RIN ≤ 10, 1.2 ≤ 28 S/16 S ≤ 2.3) was completed by Majorbio Biopharm Technology Co., Ltd., Shanghai, China. Based on the Illumina NovaSeq 6000 sequencing platform, all the mRNA isolated from the breast muscle was sequenced. The Illumina TruSeqTM RNA Sample Prep Kit was used to construct the library in the sequencing experiment. Using magnetic beads with oligo (dT) to pair with poly A, the mRNA was separated from the total RNA, and then the mRNA was fragmented and screened by magnetic beads to separate small fragments of approximately 300 bp. Double-stranded cDNA was synthesized by reverse transcriptase using mRNA as a template. Then, the adaptor was linked, and PCR amplification was used for library enrichment. A 2% agarose gel (Solarbio, Beijing, China) was used to recover the target band, and bridge PCR amplification was used to generate clusters and sequence them on the computer.

### 2.3. Quality Evaluation and Alignment Analysis

The software (fastx_toolkit_0.0.14) was used to evaluate the quality of the original sequencing data of each sample. Before the analysis, we carried out quality control on the original sequencing data to obtain high-quality quality control data (clean data) to ensure the accuracy of the follow-up analysis results by using SeqPrep (https://github.com/jstjohn/SeqPrep accessed on 18 February 2022.) and Sickle (https://github.com/najoshi/sickle accessed on 18 February 2022). The raw data after quality control, that is, clean reads, were used by TopHat2.1.1 (http://ccb.jhu.edu/software/tophat/index.shtml accessed on 25 February 2022) [14], HISAT(v2.2.0) (https://daehwankimlab.github.io/hisat2/ accessed on 5 March 2022) [15] and the reference genome (BGI_duck_1.0 Anas platyrhynchos, https://www.ncbi.nlm.nih.gov/datasets/genome/GCF_000355885.1/ accessed on 5 March 2022). Reference genome version (BGI_duck_1.0) alignment was used to obtain mapped reads for subsequent transcript assembly and expression calculation. Based on the selected reference genome sequence, the mapped reads were spliced by StringTie (http://ccb.jhu.edu/software/stringtie/ accessed on 15 March 2022) [16] to find the original unannotated transcripts and new genes of the species to supply and improve the original genome annotation information.

### 2.4. Interaction Network of DEGs

In this study, the species of duck (Anas platyrhynchos) was used as the reference data for gene comparison to construct the network. The genes in the network were visualized by Network X in Python, and the topological properties of gene interaction networks were calculated to obtain the key nodes in the interaction network. The first 300 interaction groups with a comprehensive value of 0.4 were selected to construct the network. The obtained network node information was imported into the STRING database (https://string-db.org/ accessed on 25 March 2022) for online analysis, and the nodes related to “muscle” were exported to Cytoscape (v3.7.1) [17] software for visual analysis. The connection number of the node gene degree was used to screen the key genes in the network.

### 2.5. Transcript Assembly, Differentially Expressed Gene and Enrichment Analysis

RSEM (v1.55.0) (http://deweylab.github.io/RSEM/ accessed on 27 March 2022) [18] was used to calculate gene expression, and mapped reads were used to map the number of genome annotation file sequences. Transcripts per million reads were used to compare the number of complete transcripts, and then the relative expression levels of genes were calculated. To fully explore transcriptome information, all groups were pairwise compared according to different periods of the same group and different groups of the same period. Read counts were analyzed by DESeq2 (v1.42.0) (http://bioconductor.org/packages/stats/bioc/DESeq2/ accessed on 29 March 2022) [19] based on a negative binomial distribution.

The logarithm of transcripts per million reads of gene expression was taken with 10 as the base, and the clustering among the samples was analyzed by K-means clustering. Pathway annotation and enrichment analyses were performed using the Gene Ontology (GO) and Kyoto Encyclopedia of Genes and Genomes (KEGG) pathway databases. The *p*-values less than 0.05 denoted statistical significance. The *p*-values underwent correction for multiple tests through the utilization of the Benjamini–Hochberg (BH) method.

### 2.6. Verification of Results by qRT-PCR

Total RNA was extracted from frozen muscle tissues using TRIzol reagent (Invitrogen, Delaware, DE, USA) according to the manufacturer’s instructions. RNA was reverse transcribed into cDNA by a Revert Aid™ First Strand cDNA Synthesis Kit (Thermo Fisher Scientific, Delaware, DE, USA). Each reaction was performed in triplicate using a LightCycler 96 Real-Time PCR System (Roche, Switzerland). The 20 μL reaction system included 10 μL SYBR Green Master Mix (Without ROX) (Q121-02, Vazyme, Nanjing, China), 0.4 μL forward primer (10 μM), 0.4 μL reverse primer (10 μM), 2 μL cDNA and 7.2 μL RNase-free water. The thermocycling parameters used for qRT–PCR were as follows: 95 °C for 10 min, 40 cycles at 95 °C for 10 s, 60 °C for 40 s and 95 °C for 15 s, followed by a melting curve from 60 °C for 60 s, 95 °C for 30 s and 60 °C for 15 s. *GAPDH* was used as an internal control to normalize the expression level of the target genes. All samples were repeated three times, and the mean and standard error values were calculated. Relative expression of all genes was calculated by the 2^−ΔΔCT^ method. The results were expressed as mean ± SD of at least three independent biological replicates. One-way ANOVA in SPSS 26.0 software was used to compare the significance of mean values through Duncan’s test, and significance was represented by the *p*-value; *p* < 0.05 was considered significantly different.

## 3. Results

### 3.1. Transcriptomic Screening and Analysis of Duck Breast Muscle Tissue

**Figure 1:**
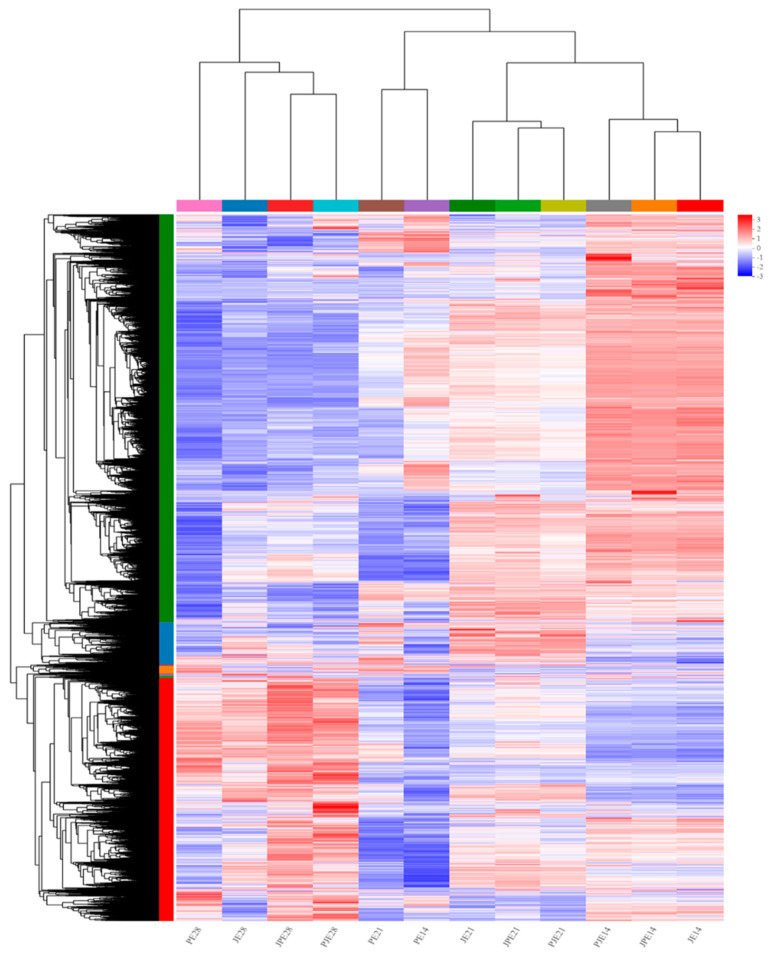
Differentially expressed gene clustering heat map, utilizing sample mean expression values from each condition. The abscissa is the sample name, and the ordinate is the normalized value of the differential gene FPKM. The color in the heat map represents gene expression changes. Red indicates upregulation of gene expression, blue indicates downregulation of expression, darker color means a remarkable degree of differential gene expression and white indicates no activity.

Transcriptome expression differences of four species of ducks (P, J, PJ and JP) at three time points were studied by RNA-seq. Total RNA was extracted from four kinds of duck breast muscle tissues at three time points, and 36 cDNA libraries (three time points in each group, three samples at each time point) were sequenced using the Illumina HiSeq platform. A total of 307.91 Gb of raw reads and 297.59 Gb of clean reads were obtained in this experiment. All of the raw sequence data have been deposited in the National Center for Biotechnology Information (NCBI) Sequence Read Archive (SRA) (https://www.ncbi.nlm.nih.gov/sra, accessed on 20 December 2023) under accession number PRJNA1056445. The clean reads of each sample after filtration were more than 6.26 Gb, with Q30 ≥ 94.39% and GC content ranging from 50.12% to 54.27%. Clean reads were compared separately to the duck genome, and the total mapped reads were between 60.76% and 76.6% (Table 1). The above results indicated that the three biological samples from each group at various periods presented similar high performance, the relevant experimental sampling procedures and experimental conditions met the requirements of sequencing and the results could be used for subsequent bioinformatics analysis. To thoroughly investigate the transcriptomic differences among groups and across different time points, DESeq2(v1.42.0) software was utilized. A significance threshold of *p*-adjust < 0.05 and |log_2_FC| ≥ 1 was applied. The hierarchical clustering method was used to compare the DEGs of different duck species at different periods (14, 21 and 28 days), and a gradual change in gene expression was observed (Figure 1). DEGs could be further classified on the left side of the hierarchical clustering gene tree, where the gene expression pattern was similar among groups at 28 days of embryonic stage, and the PJ and JP groups were closest. The J, PJ and JP gene expression patterns at the 14- and 21-day embryonic stage were similar overall, but the clustering of the 14- and 21-day embryonic stage was closer in Group P, indicating that the gene expression patterns at early embryonic development of Pekin ducks were significantly different from those of the other three breeds.

### 3.2. Protein-Protein Interaction Network Predictive Analysis

**Figure 2:**
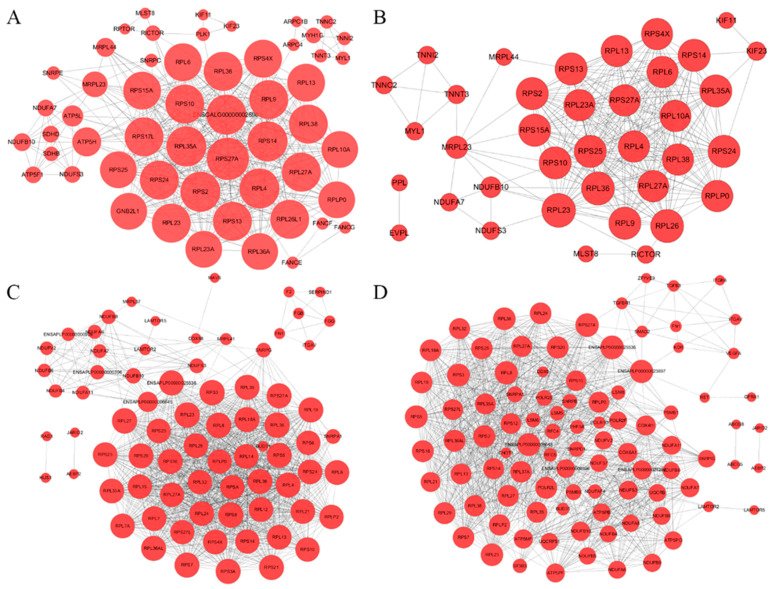
Protein–protein interaction (PPI) network of the differentially expressed genes (DEGs). (**A**) Interaction network of all DEGs. (**B**) Interaction network of DEGs at day 14. (**C**) Interaction network of DEGs at day 21. (**D**) Interaction network of DEGs at day 28.

A total of 5053 DEGs (Figure S1A) obtained from the comparison between different groups at each time point were used as one gene set for protein interaction analysis and prediction on the Megi Biocloud platform (Figure 2A). Protein interaction network prediction analysis was also performed on the DEGs obtained from the comparison of different groups at 14, 21 and 28 days, and the number of DEGs at the three time points was 3006 (E14), 2439 (E21) and 2876 (E28), respectively (Figure S1B–D). A total of three protein interaction network prediction diagrams were obtained, as shown in Figure 2B–D. The largest common part of the protein interaction network prediction diagram was ribosome-related genes, such as *RPS27A*, *RPS25* and *RPL4*, followed by genes in oxidative phosphorylation pathways, such as *NUDFA7*, *NUDFB10* and *NUDFS3* (Figure 2A). In addition, myosin-related genes in fast muscle fibers, such as *TNNI2*, *TNNC2*, *TNNT3* and *MYL1*, were present at 14 days of the embryonic stage (Figure 2B), indicating that large-scale synthesis of muscle fibers was involved in the early stages of the embryonic stage.

### 3.3. DEG Screening for the Early Development of Ducks

**Figure 3:**
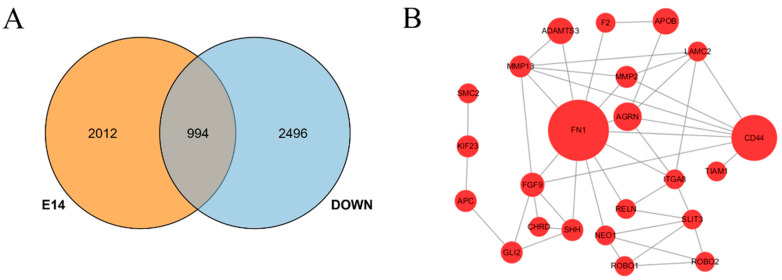
Differentially expressed genes (DEGs) in early development of duck embryos. (**A**) A Venn diagram showed the relationships among DEGs in day 14 and down-regulated DEGs in red module. (**B**) Interaction network diagram of intersection genes in A; only nodes with Degree ≥ 8 are displayed.

There were two differential gene expression patterns in duck embryos, namely, the blue module gene, which was upregulated, and the red module gene, which was downregulated (Figure S2). After gene functional enrichment analysis, it was found that, compared with the blue module, the genes of the red module were mostly enriched in muscle-related GO entries and KEGG signaling pathways (Tables S1 and S2). Studies have shown that the early embryo is a critical period for muscle growth and development. In this experiment, the number of DEGs among the groups at 14 days of embryonic age was the highest, and the interaction with genes or proteins related to muscle development was the strongest (Tables S3–S6). We conducted a second comparative analysis of the DEGs obtained by comparison among 14-day-old groups and the red module DEGs obtained by STEM clustering and found 994 common DEGs (Figure 3A and Figure S3). We also compared these common DEGs with the STRING database to obtain the gene interaction network diagram of degree > 8 (Figure 3B). Among them, *FN1*, *CD44*, *AGRN*, *ADAMTS3*, *APOB* and *FGF9* had the highest correlation, and their degrees were 27, 20, 12, 11, 11 and 10, respectively.

### 3.4. GO Enrichment and KEGG Function Analysis

**Figure 4:**
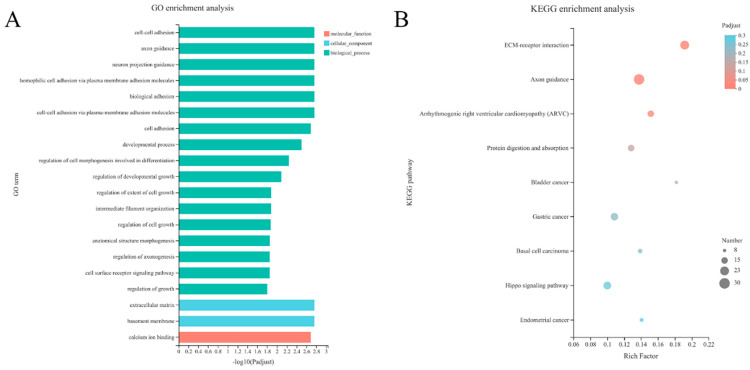
Functional enrichment results of key candidate genes. (**A**) Statistical chart of the top 20 items with significant enrichment of gene ontology (GO) function. Red, blue and green represent GO items enriched related to molecular function, cell composition and biological process, respectively. (**B**) Bubble diagram of Kyoto Encyclopedia of Genes and Genomes (KEGG) function enrichment. KEGG path shows the signal path name. Rich factor shows the ratio of the number of genes in the pathway to the number of annotated genes, and its size is positively correlated with the enrichment degree. Bubble size indicates that the number of genes and color indicate the corresponding significance.

To analyze the functions of all DEGs progressively downregulated at 14 days of embryonic age, the 994 DEGs screened in Figure 3A were analyzed for GO functional enrichment, among which the first 20 GO entries significantly enriched are shown (Figure 4A, Table S7). There were 17 items in the molecular function category, 2 items in the cell composition category, and 1 item in the biological process category. In addition, cell–cell adhesion was the most significant biological process, with 26 genes enriched. The extracellular matrix was the most significant GO item in cell composition, with a total of 37 genes. Among the molecular function category, the most significant item was calcium ion binding, and 66 genes were significantly enriched. Key candidate genes downregulated at 14 days of embryonic age were significantly enriched in three KEGG signaling pathways, including extracellular matrix (ECM) receptor interaction, axon orientation and right ventricular arrhythmia myopathy signaling pathways (Figure 4B, Table S8).

### 3.5. Validation of RNA-Seq Data Using qRT-PCR

**Figure 5:**
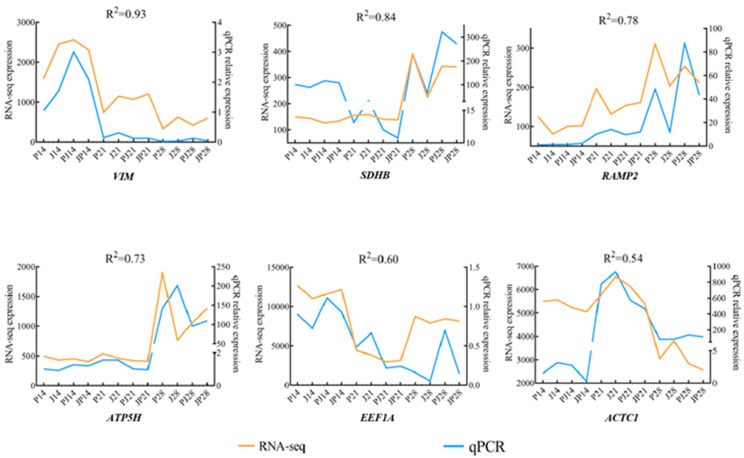
Validation of the RNA transcriptome sequencing (RNA-seq) results by qRT-PCR. Expression profiles of selected genes, including *VIM*, *SDHB*, *RAMP2*, *ATP5H*, *EEF1A* and *ACTC1*, that were involved in viral infection, as determined by qRT-PCR. *GAPDH* was used for normalization. The *x*-axis represents differentially expressed gene (DEG) expression time, whereas the left and right sides of the *y*-axis represent the TPM (Transcripts per million) value of RNA-seq and the relative gene expression, respectively.

Following RNA-seq analysis, six genes, including *VIM*, *SDHB*, *RAMP2*, *ATP5H*, *EEF1A* and *ACTC16*, were selected randomly for further validation with qRT–PCR. Primer information is shown in Table S9. qRT–PCR analysis data showed that most trends for the selected six genes corroborated the results from RNA-seq analysis. Among them, *VIM* had the highest correlation of 0.93, and *ACTC1* had the lowest correlation of 0.54 (Figure 5). The above tests verified the accuracy and reliability of the RNA-seq analysis results.

## 4. Discussion

As an important local bird species in Beijing, the Pekin duck is raised worldwide because of its delicious meat and fast growth rate [20]. In this study, Jinling White ducks were obtained by crossbreeding between purebred Liancheng White ducks (female parent) and Cherry Valley ducks (male duck), with typical features such as black beak, white feathers and black feet. The hybrid duck seems to be a potential breed of meat duck owing to its good characteristics inherited from the Pekin duck and Jinling White duck. Research has revealed variations in the expression levels of fatty acid-related genes in the breast muscles of different duck strains, resulting in diverse fatty acid compositions and consequently influencing the quality of the breast muscle [21]. Therefore, the selection of superior varieties is pivotal in optimizing livestock production.

In order to study the differences in skeletal muscle development between the hybrid ducks and their fathers and mothers during the embryonic period, the breast muscles of 14 days, 21 days and 28 days were selected for transcriptome sequencing analysis. Ducks have an incubation period of 28 days, but muscle development begins at 7 days when the muscle tissue is not fully formed and cannot be accurately sampled [22]. In addition, studies have shown that the embryonic development of ducks can be divided into three general stages: 10–17 days is the transition period from mononuclear-muscle-fiber formation to muscle-fiber fusion, 18–23 days is the stage of muscle-tube fusion and muscle-bundle structure formation and 24–28 days is the stage of muscle-fiber maturation. On the other hand, the breast muscle, which developed earlier and faster than the leg muscle in the embryonic stage, had a relatively simple muscle-fiber type, and the sampling convenience was selected as the test tissue [5,23]. Therefore, three time points at the same interval were selected to comprehensively understand the differences in skeletal muscle development of ducks in different populations.

To investigate the variations in embryonic skeletal muscle development between hybridization and parent breeds, four distinct groups of ducks were established in this study. The genes acquired from each group at different time points were used as the threshold for detecting differential doubling, and pairwise comparisons were conducted to identify differentially expressed genes. Subsequently, these genes were subjected to hierarchical clustering analysis, revealing distinct expression patterns in the pectoral muscle transcriptome among the duck groups. Notably, significant differences were observed between Beijing ducks and the other three groups, suggesting that despite Beijing ducks exhibiting rapid growth, the genetic dominance of Jinling White ducks may exert a more substantial influence on offspring muscle development. The skeletal muscle embryonic development of the Jinling White duck, as a new strain bred in local ducks, is still far from that of Pekin ducks, which is consistent with the results of studies on Pekin ducks and other ducks [23,24].

Skeletal muscle, which makes up 40% to 60% of the body’s total weight, is an important tissue involved in regulating the metabolism of glucose and lipids, as well as movement and strength [25,26]. The skeletal muscle of poultry undergoes structural development and functional maturation during incubation. For example, myoblasts proliferate and differentiate into multinucleated muscle tubes and eventually become mature muscle fibers. However, the total number of muscle fibers was fixed during the final stages of avian embryonic development, so the early embryo played an important role in skeletal muscle development [27,28,29,30].

There are many complex regulatory models involved in the progressive development of skeletal muscle, which require a large number of genes and transcription factors to cooperate in each stage of development [31,32]. For example, the *myog*enic regulatory factor family (*Myf5*, *Myf6*, *MyoD* and *MyoG*), *MEF2* gene family, *Pax* gene family and insulin growth-like factor (*IGF*) family are involved in the development and differentiation of various types of cells during embryogenesis [33,34]. In this study, the most common part of the network diagram was ribosome-related genes, followed by genes in oxidative phosphorylation pathways (Figure 2). In addition, at 14 days in the embryo, a large number of troponin-related genes, such as *TNNI2*, *TNNC2*, *TNNT3* and *MYL1*, were found to be expressed in muscle fibers. Three subunits (*TNNC*, *TNNT* and *TNNI*) are known to form troponin [35]. These results indicated that proteins related to muscle development were formed in the early stages of the embryo. At this stage, skeletal muscle-related functions gradually mature through the cooperation of multiple gene targets.

Transcriptome analysis is a powerful tool for investigating the gene function of ducks. RNA-seq implicated candidate genes, such as *P2RX1*, which influence the laying rate of Muscovy ducks [25]. In this study, the breast muscle tissues of Pekin ducks, Jinling White ducks and their cross-crossing progeny were collected at Days 14, 21 and 28 of embryonic life, and transcriptome sequencing was performed to compare the differences to reveal the key genes and signaling pathways that played a role in the early growth and development of ducks. The results showed that 994 differentially expressed genes were obtained by comparing the differentially expressed genes and STEM clustering of all differentially expressed genes in red modules. KEGG and GO analyses further revealed key genes and pathways involved in the early embryonic development of ducks, including intercellular adhesion, extracellular matrix, calcium ion binding, ECM receptor interactions, axon steering and right ventricular arrhythmia myopathy.

At the 14 days of embryonic development, we found the highest count of differentially expressed genes among the groups with the most robust interaction observed with genes or proteins associated with muscle development. Notably, the gene expression pattern in the breast muscle of duck embryos at this stage mirrors findings from prior studies, emphasizing the pivotal role of the early embryo period in muscle growth and development. Consequently, through thorough screening and comparison using two distinct approaches, we designated 14 days of duck embryo development as a specific timeframe for investigating differences in muscle development across the four groups in this study.

During the 14 days of duck embryonic development, 994 DEGs were screened. By comparison with the STRING database, 6 DEGs (*FN1*, *CD44*, *AGRN*, *ADAMTS3*, *APOB* and *FGF9*) had the highest correlation degree. *FN1* is a macromolecular glycoprotein that widely exists in tissues and tissue fluids and is mainly involved in adhesion between cells and the matrix, as well as between cells [36,37]. Studies have shown that *FN1* is also involved in epithelial tissue migration of myocardial progenitor cells, cardiovascular formation and mesoderm development during the embryonic period of animals [38,39]. In this study, the expression level of *FN1* in the four groups showed a decreasing trend with increasing embryo age and even significantly decreased after 21 days of embryonic development, suggesting that *FN1* plays a crucial role in the development of skeletal muscle in early embryos. *FN1* overexpression induces transforming growth factor-β (*TGF-β*) signaling activation [40]. The *TGF-β* family contains a variety of proteins, the most important of which is myostatin. Myostatin induces Smad2/3 complex formation by activating downstream Smad proteins, which suppress *MyoD* expression and arrest myoblasts in G1 and G2 phases, thereby inhibiting myoblast proliferation, differentiation and fusion [41]. Therefore, reduced *FN1* expression in duck embryonic breast muscle may downregulate the *TGF-β* family member myostatin, which alleviates its inhibition of *MyoD* and thus facilitates muscle development. A whole-genome sequencing (WGS) study also conducted in sheep suggests the involvement of the differentially methylated gene *FN1* in muscle development [42].

In addition, this study showed, for the first time, that *CD44* could play an important role in the early development of duck embryos. As a single-chain transmembrane glycoprotein, *CD44* is widely involved in biological processes, such as cell interaction, adhesion, hematopoietic and tumor metastasis [43]. A mouse study showed that *CD44* was highly expressed in the embryonic stage and mediated muscle development by binding to its ligand HA, activating downstream signaling, interacting with fibronectin in the ECM of myofibroblasts and enhancing cell migration and proliferation. Blocking *CD44* synthesis reduced the forelimb muscle mass of mice. Interestingly, *TGF-β* also regulated *CD44*, which was highly expressed in the duck embryonic stage [44,45]. Therefore, we hypothesized that *CD44* might be a key gene for inducing muscle development in duck embryos.

*AGRN* mutations can induce congenital myasthenia and affect synaptic function and cardiomyocyte regeneration [46,47].

Studies have reported that *ADAMTS3* might be associated with embryonic development in mammals [48,49]. The *ADAMTS3/VEGF-C/VEGF-R3* axis is essential for the development of embryonic lymphatic and placental vessels. Reduced *ADAMTS3* expression leads to a thinner embryonic labyrinthine layer and impaired oxygen delivery, which affects embryonic development. Thus, *ADAMTS3* might indirectly modulate embryonic muscle development by influencing the vascularization of duck embryonic muscle tissue.

*APOB* usually acts on energy transport and metabolism and participates in abdominal fat deposition and body development [50,51]. *FGF9* is an important member of the fibroblast growth factor family that promotes the proliferation of epithelial cells, glial cells and fibroblasts, as well as the development of the embryonic reproductive system and lung tissue [52,53]. Other studies have shown that *FGF9* not only acts on the specific differentiation of skeletal muscle but also inhibits *myog*enic differentiation of C2C12 cell lines by downregulating *myog*enic regulatory factor (*Myog*enim) [54]. Therefore, the six genes identified in this study might play a crucial role in the development of breast muscle in duck embryos. Among the four duck breeds investigated in this experiment, Beijing ducks exhibited the fastest rate of muscle development, while Jinling ducks displayed the slowest. Additionally, the JP group showed a slightly higher rate compared to the PJ group. The observed phenotype differences were attributed, in part, to different expressed genes. To elucidate these differences, we conducted a comprehensive comparison of the various groups and identified and enriched all differential genes through GO and KEGG analyses.

Through GO and KEGG analysis, we found significant enrichment of ECM, which plays an important role in regulating the development, function and homeostasis of eukaryotic cells and is also involved in the processes of cell differentiation and migration [55,56,57]. Other studies suggested that the highly arranged collagen bundles of ECM in muscle tissue were added to the C2C12 cell medium for culture, and the results showed that the expression of *MyoG*, *MyoD* and *Myf5* was upregulated, indicating that ECM promoted collagen cell differentiation and maturation [58]. In addition, we found that a total of 19 genes were enriched in the hippo signaling pathway during the KEGG pathway analysis. The hippo signaling pathway is usually involved in biological events, such as cell proliferation, cell death, cell differentiation, organ size regulation and cancer occurrence [59]. Our study indicated that the hippo signaling pathway might be involved in early skeletal muscle development in duck embryos.

## 5. Conclusions

In this study, the transcriptome of breast muscle tissue of four breeds of ducks was studied based on RNA-Seq technology, and candidate genes and signaling pathways related to skeletal muscle traits were screened and identified. Network interaction prediction analysis among proteins showed that there were 3006, 2439 and 2878 DEGs at 14, 21 and 28 days of age, respectively. Among them, 14 days of embryonic age played an important role in embryonic development, and *FN1*, *CD44*, *AGRN*, *ADAMTS3*, *APOB* and *FGF9* might be candidate genes for regulating muscle development. In addition, GO analysis showed functional enrichment in molecular function, cell components and biological processes, while KEGG analysis revealed that the hippo pathway and ECM receptor action pathway could be vital pathways regulating skeletal muscle during 14-day-old embryonic development.

## Figures and Tables

**Table 1:** Summary of Illumina RNA-seq data.

Group ^1^	Sample	Clean Reads ^2^	Clean Bases (Gb) ^3^	Q20 (%) ^4^	Q30 (%) ^5^	GC Content (%) ^6^
J14	1	54,964,154	8.15	98.16	94.76	52.32
	2	66,766,480	9.89	98.19	94.85	52.47
	3	55,963,296	8.28	98.23	94.91	52.24
J21	1	49,696,672	7.27	98.29	94.96	50.12
	2	59,501,280	8.80	98.15	94.77	53.51
	3	63,014,954	9.32	98.16	94.80	53.69
J28	1	66,050,398	9.78	98.20	94.89	54.06
	2	61,710,802	9.14	98.11	94.67	54.11
	3	62,580,976	9.26	98.22	94.92	53.48
JP14	1	58,135,548	8.61	98.11	94.66	53.24
	2	59,868,580	8.90	98.18	94.82	52.97
	3	55,533,882	8.25	98.06	94.56	52.43
JP21	1	54,631,272	8.08	98.12	94.72	52.82
	2	61,418,642	9.11	98.08	94.60	53.06
	3	58,556,596	8.68	98.15	94.68	52.89
JP28	1	58,369,278	8.67	98.17	94.79	52.64
	2	53,300,948	7.91	98.09	94.59	52.49
	3	56,088,598	8.33	98.09	94.59	52.74
P14	1	46,643,606	6.90	98.10	94.66	52.85
	2	49,462,680	7.28	98.23	95.03	53.73
	3	57,653,512	8.53	98.07	94.60	53.78
P21	1	54,928,900	8.12	98.02	94.46	53.18
	2	49,144,136	7.26	98.07	94.60	54.26
	3	46,298,258	6.84	97.97	94.45	54.26
P28	1	52,808,292	7.80	98.09	94.66	53.29
	2	49,559,050	7.31	97.99	94.39	53.52
	3	42,368,078	6.27	98.06	94.63	53.80
PJ14	1	49,781,428	7.39	98.07	94.65	52.82
	2	57,148,852	8.47	98.10	94.59	52.48
	3	56,494,950	8.41	98.16	94.73	52.80
PJ21	1	60,868,366	9.06	98.14	94.71	53.55
	2	58,061,018	8.62	98.00	94.43	53.52
	3	58,194,764	8.63	98.20	94.88	52.65
PJ28	1	55,547,062	8.22	98.16	94.82	54.27
	2	52,360,940	7.75	98.19	94.87	53.51
	3	56,167,902	8.30	98.13	94.76	52.69

^1^ J represents Jinling White duck breeding group; JP represents Jinling White duck ♂ × Peking duck ♀. In the reciprocal-cross group, P represents Pekin duck pure breeding group; PJ represents Jinling White duck ♂ × Peking duck ♀. In the orthogonal group, 14, 21 and 28 represent samples collected 14, 21 and 28 days after hatching. ^2^ The number of reads after filtering the original data. ^3^ The number of bases after filtering the original data. ^4^ The percentage of bases with a Phred value greater than 20 to the total bases. ^5^ The percentage of bases with a Phred value greater than 30 to the total bases. ^6^ The percentage of G and C in the four bases in clean reads.

## Data Availability

Data from this study not included in the article may be obtained from the corresponding author upon reasonable request.

## Supplementary Materials

The following supporting information can be downloaded at: https://www.mdpi.com/article/10.3390/genes15010052/s1, Figure S1: (A) Venn diagram of DEGs in different stages of each breed. (B) Venn diagram of DEGs in 14 days of each breed. (C) Venn diagram of DEGs in 21 days of each breed. (D) Venn diagram of DEGs in 28 days of each breed. Figure S2: (A) STEM cluster analysis of DEGs at different growth stages in the Pekin duck pure breeding group (P). (B) STEM cluster analysis of DEGs at different growth stages in Jinling White duck breeding group (J). (C) STEM cluster analysis of DEGs at different growth stages in Pekin duck ♂ × Jinling White duck ♀ orthogonal group (PJ). (D) STEM cluster analysis of DEGs at different growth stages in Jinling White duck ♂ × Peking duck ♀ reciprocal-cross group (JP). The broken line in the rectangle was trend variation. Figure S3: (A) Red module DEGs obtained by STEM clustering. (B) Blue module DEGs obtained by STEM clustering. Table S1: Red module genes are enriched into the GO entries related to muscle description. Table S2: The GO enrichment of blue module terms significantly related to muscle. Table S3: KEGG pathway information with significantly enriched differentially expressed genes between Peking duck and Jinling white duck in each period. Table S4: Kyoto Encyclopedia of Genes and Genomes (KEGG) significant enrichment pathway of key candidate genes. Table S5: Top 10 pathways in which differentially expressed genes between Peking duck and backcross duck were significantly enriched by KEGG in each period. Table S6: KEGG pathway information with significantly enriched differentially expressed genes between Jinling white duck and backcross duck in each period. Table S7: Top 20 entries of the GO functional enrichment of key candidate genes. Table S8: KEGG significant enrichment pathway of key candidate genes. Table S9: qRT–PCR Primers List.
