# Supplementary material for: Transcriptome Profiling Identifies Differentially Expressed Genes in Skeletal Muscle Development in Native Chinese Ducks
Source: Genes (Basel). 2023 Dec 28;15(1):52. doi: 10.3390/genes15010052 (PMC10815232; doi:10.3390/genes15010052)
Supplement: Supplementary file 1 [file genes-15-00052-s001.zip › genes-2724776-supplementary.pdf]

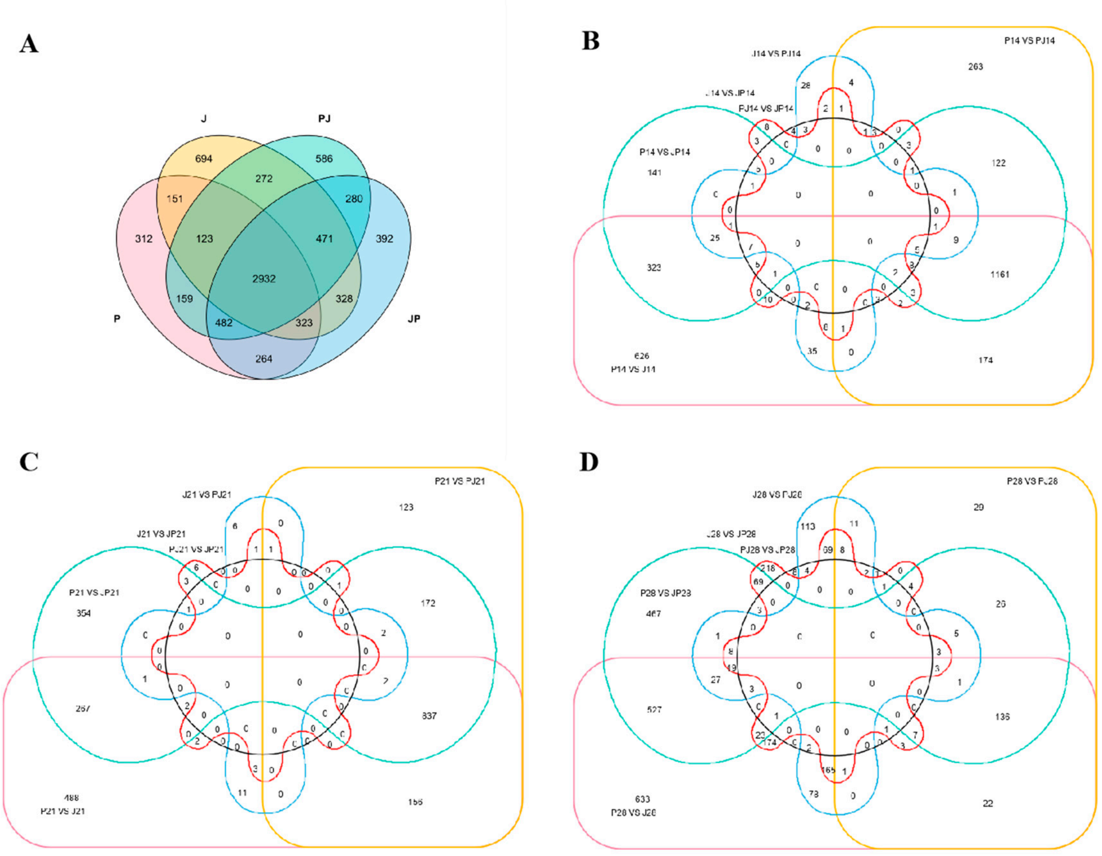

Figure S1. (A) Venn diagram of differentially expressed genes (DEGs) in different stages of each breed. (B) Venn diagram of DEGs in 14 days of each breed. (C) Venn diagram of DEGs in 21 days of each breed. (D) Venn diagram of DEGs in 28 days of each breed.

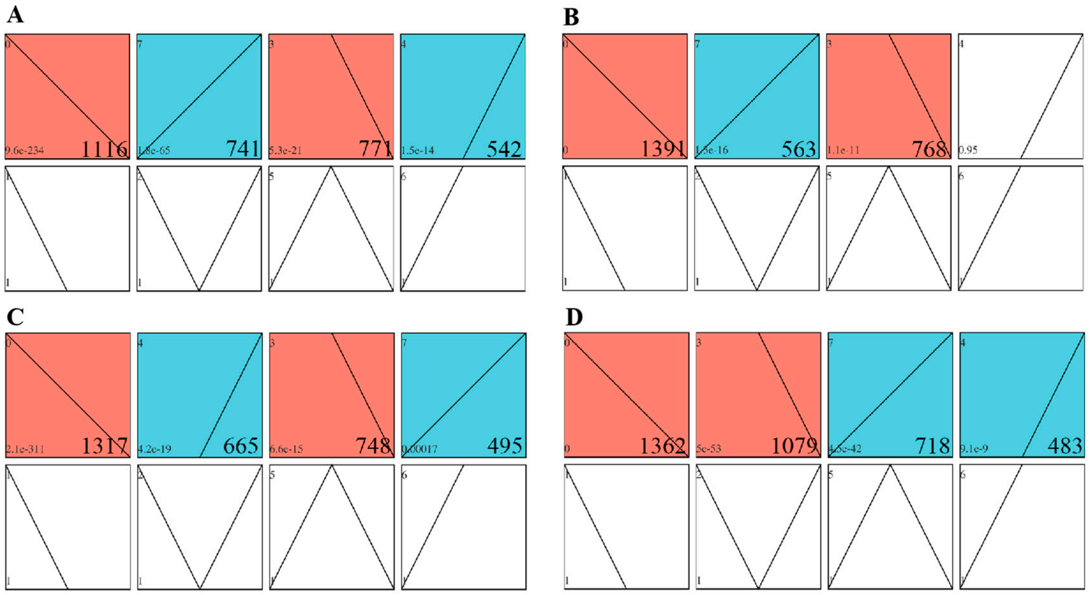

Figure S2. (A) STEM cluster analysis of differentially expressed genes (DEGs) at different growth stages the Pekin duck pure breeding group (P). (B) STEM cluster analysis of DEGs at different growth stages in Jinling White duck breeding group (J). (C) STEM cluster analysis of DEGs at different growth stages in Pekin duck ♂ × Jinling White duck ♀ orthogonal group (PJ). (D) STEM cluster analysis of DEGs at different growth stages in Jinling White duck ♂ × Peking duck ♀ reciprocal-cross group (JP). The broken line in the rectangle was trend variation. The number in the upper left corner represents the number of the corresponding expression pattern, the value in the lower left corner was the gene enrichment to the significance level (*P*-value) of the module, and the number of genes in the module was in the lower right corner. Trend map with color shows that the timing mode of the profile was consistent with the trend of significant change. The profiles with the same color represents the same cluster (profiles with similar trend are classified as one class). Trend chart without color: the timing pattern of the profile was statistical nonsignificant trend. The expression patterns were as follows: expression pattern 0 (the expression of each gene decreased gradually), expression pattern 1 (the expression of each gene decreased first and then did not change

significantly), expression pattern 2 (the expression of each gene decreased first and then increased), expression pattern 3 (the expression of each gene did not change significantly and then decreased), expression pattern 4 (the expression of each gene did not change significantly and then increased) Expression pattern 5 (the expression of each gene increased first and then decreased), expression pattern 6 (the expression of each gene increased first and then did not change significantly), expression pattern 7 (the expression of each gene increased gradually).

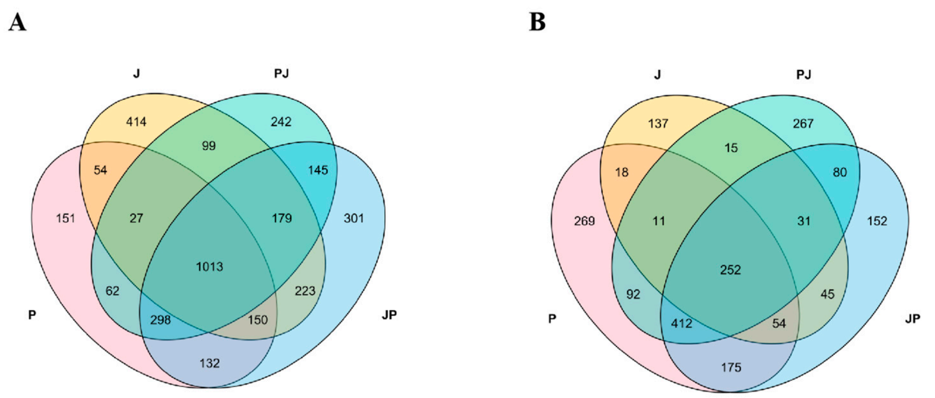

Figure S3. (A) Red module differentially expressed genes (DEGs) obtained by STEM clustering. (B) Blue module DEGs obtained by STEM clustering.

Table S1. Red module genes are enriched into the Gene Ontology (GO) entries related to muscle description

Gene Number	GO ID	Description	*P*-value	*P*-adjust
4	GO:0043403	skeletal muscle tissue regeneration	0.001359	0.104832
2	GO:1902809	regulation of skeletal muscle fiber differentiation	0.036896	0.498255
2	GO:0086042	cardiac muscle cell-cardiac muscle cell adhesion	0.036896	0.498255
2	GO:0098911	regulation of ventricular cardiac muscle cell action potential	0.036896	0.498255
11	GO:0016202	regulation of striated muscle tissue development	0.040572	0.540377
8	GO:0042692	muscle cell differentiation	0.045836	0.587429
11	GO:0048634	regulation of muscle organ development	0.046737	0.589675
4	GO:0014857	regulation of skeletal muscle cell proliferation	0.049274	0.589675

Table S2. The Gene Ontology (GO) enrichment of blue module terms significantly related to muscle

Gene Number	GO ID	Description	*P*-value	*P*-adjust
15	GO:0003012	muscle system process	0.000116128	0.011257803
8	GO:0061061	muscle structure development	0.00027111	0.020948484
8	GO:0007517	muscle organ development	0.00027111	0.020948484

Table S3. KEGG pathway information with significantly enriched differentially expressed genes between Peking duck and Jinling white duck in each period

Comparisons	Pathway ID	Gene Number	Description	P-adjust
PE14 VS JE14	map03010	77	Ribosome	1.81666E-27
	map00190	32	Oxidative phosphorylation	0.005208054
	map04512	31	ECM-receptor interaction	0.017456737
PE21 VS JE21	map03010	67	Ribosome	1.06214E-26
	map00190	29	Oxidative phosphorylation	0.000335655
	map04714	43	Thermogenesis	0.002483961
	map05012	27	Parkinson disease	0.016310537
PE28 VS JE28	map00190	57	Oxidative phosphorylation	2.55838E-24
	map05012	55	Parkinson disease	2.24282E-19
	map04714	73	Thermogenesis	2.88828E-18
	map04932	46	Non-alcoholic fatty liver disease (NAFLD)	3.03508E-11
	map05016	70	Huntington disease	4.99436E-11
	map05010	79	Alzheimer disease	1.34107E-10
	map04512	35	ECM-receptor interaction	1.75019E-07
	map04723	35	Retrograde endocannabinoid signaling	7.40067E-05
	map04260	26	Cardiac muscle contraction	0.000268255
	map04510	46	Focal adhesion	0.00079965

Table S4. KEGG pathway information with significantly enriched differentially expressed genes between Peking duck and orthogonal duck in each period

Comparisons	Pathway ID	Gene Number	Description	P-adjust
PE14 VS PJE14	map03010	81	Ribosome	2.05748E-40
	map00190	36	Oxidative phosphorylation	4.06616E-08
	map05012	34	Parkinson disease	1.53698E-05
	map04714	46	Thermogenesis	0.000165049
	map04932	33	Non-alcoholic fatty liver disease (NAFLD)	0.000238831
	map04510	45	Focal adhesion	0.001875767
	map04512	27	ECM-receptor interaction	0.002102611
	map04151	61	PI3K-Akt signaling pathway	0.006613725
	map05219	13	Bladder cancer	0.008779746
	map05410	25	Hypertrophic cardiomyopathy (HCM)	0.01088043
PE21 VS PJE21	map03010	69	Ribosome	5.50926E-37
	map04512	28	ECM-receptor interaction	4.4122E-06
	map00190	21	Oxidative phosphorylation	0.009630652
	map04510	34	Focal adhesion	0.019044239
	map05410	20	Hypertrophic cardiomyopathy (HCM)	0.036744269
PE28 VS PJE28	map04150	11	mTOR signaling pathway	0.006138745
	map04923	7	Regulation of lipolysis in adipocytes	0.011236781
	map04960	5	Aldosterone-regulated sodium reabsorption	0.033832119

Table S5. Top 10 pathways in which differentially expressed genes between Peking duck and backcross duck were significantly enriched by KEGG in each period

Comparisons	Pathway ID	Gene Number	Description	P-adjust
PE14 VS JPE14	map03010	77	Ribosome	2.08528E-35
	map00190	34	Oxidative phosphorylation	1.319E-06
	map04512	33	ECM-receptor interaction	8.03584E-06
	map05010	61	Alzheimer disease	0.001017494
	map04510	47	Focal adhesion	0.001096359
	map05219	15	Bladder cancer	0.00136205
	map04932	31	Non-alcoholic fatty liver disease (NAFLD)	0.002077485
	map05012	29	Parkinson disease	0.002143528
	map04714	42	Thermogenesis	0.003600948
	map05165	61	Human papillomavirus infection	0.003824928
PE21 VS JPE21	map03010	83	Ribosome	8.86918E-45
	map00190	34	Oxidative phosphorylation	1.25346E-07
	map04714	46	Thermogenesis	3.58377E-05
	map05012	31	Parkinson disease	8.6821E-05
	map05016	49	Huntington disease	0.001201827
	map04932	29	Non-alcoholic fatty liver disease (NAFLD)	0.002909412
	map04512	25	ECM-receptor interaction	0.00450946
	map05010	54	Alzheimer disease	0.005600459
	map05219	12	Bladder cancer	0.018564395
PE28 VS JPE28	map03010	70	Ribosome	6.05923E-38
	map00190	34	Oxidative phosphorylation	1.80535E-10
	map04714	46	Thermogenesis	2.15214E-

			08
map05012	31	Parkinson disease	3.83902E-07
map04932	29	Non-alcoholic fatty liver disease (NAFLD)	3.32291E-05
map05016	40	Huntington disease	0.002961745
map05010	43	Alzheimer disease	0.025856558

Table S6. KEGG pathway information with significantly enriched differentially expressed genes between Jinling white duck and backcross duck in each period

Comparisons	Pathway ID	Gene Number	Description	P-adjust
JE28 VS JPE28	map04512	22	ECM-receptor interaction	3.11343E-06
	map00260	9	Glycine, serine and threonine metabolism	0.021895724
	map04974	14	Protein digestion and absorption	0.030063718
	map05410	15	Hypertrophic cardiomyopathy (HCM)	0.03159392
	map04933	15	AGE-RAGE signaling pathway in diabetic complications	0.03159392
	map00270	9	Cysteine and methionine metabolism	0.035558336

Table S7. Top 20 entries of the Gene Ontology (GO) functional enrichment of key candidate genes

Gene Number	GO ID	Description	*P*-value	*P*-adjust
26	GO:0098609	cell-cell adhesion	4.37238E-07	0.001735577
18	GO:0007411	axon guidance	4.61042E-07	0.001735577
19	GO:0097485	neuron projection guidance	5.8386E-07	0.001735577
17	GO:0007156	homophilic cell adhesion via plasma membrane adhesion molecules	9.15518E-07	0.001735577
39	GO:0022610	biological adhesion	9.98578E-07	0.001735577
17	GO:0098742	cell-cell adhesion via plasma-membrane adhesion molecules	1.04224E-06	0.001735577
37	GO:0031012	extracellular matrix	6.13622E-07	0.001735577
9	GO:0005604	basement membrane	1.15091E-06	0.001735577
39	GO:0007155	cell adhesion	1.92962E-06	0.002047445
66	GO:0005509	calcium ion binding	2.04492E-06	0.002047445
109	GO:0032502	developmental process	3.49611E-06	0.003163284
14	GO:0010769	regulation of cell morphogenesis involved in differentiation	7.59478E-06	0.005726462
16	GO:0048638	regulation of developmental growth	1.14567E-05	0.008183692
9	GO:0061387	regulation of extent of cell growth	2.22284E-05	0.013116678
5	GO:0045109	intermediate filament organization	2.43327E-05	0.013209756
17	GO:0001558	regulation of cell growth	2.56219E-05	0.013374609
41	GO:0009653	anatomical structure morphogenesis	2.79205E-05	0.014034688
11	GO:0050770	regulation of axonogenesis	2.9628E-05	0.014048039
60	GO:0007166	cell surface receptor signaling pathway	3.00172E-05	0.014048039
22	GO:0040008	regulation of growth	3.59843E-05	0.015800202

Table S8. Kyoto Encyclopedia of Genes and Genomes (KEGG) significant enrichment pathway of key candidate genes

Gene Number	Pathway ID	Description	P-value	P-adjust
23	map04512	ECM-receptor interaction	1.09497E-07	3.33965E-05
30	map04360	Axon guidance	2.63691E-06	0.000402129
15	map05412	Arrhythmogenic right ventricular cardiomyopathy (ARVC)	0.00028822	0.029302389
15	map04974	Protein digestion and absorption	0.001705554	0.130048487
8	map05219	Bladder cancer	0.002316865	0.141328773
18	map05226	Gastric cancer	0.004125073	0.209691188
10	map05217	Basal cell carcinoma	0.005453188	0.23760318
19	map04390	Hippo signaling pathway	0.007797887	0.264261725
9	map05213	Endometrial cancer	0.007551891	0.287915849

Table S9. Validation of DEGs by qRT-PCR

Gene name	GenBank accession	Primer sequence (5′-3′)	Product size (bp)
VIM	XM_027450688.2	Forward: TGCCCTTAAAGGAAGCAATG Reverse: CTCCCTCCAGCAGTTTTCTG	227
SDHB	XM_027443169.2	Forward: CCCATGGTACTCGATGCTCT Reverse: TCCGGAACAAGATCCTTCAC	218
RAMP2	XM_038168767.1	Forward: TCGGTGGGCAACCAGGTGTT Reverse: TCCTTGCTGCGCCAGATGAC	175
ATP5H	XM_027471379.2	Forward: CAGTGGACACGCAAACTGCT Reverse: CAGCCATGTCTTCGTTTGTCA	168
EEF1A	XM_038175376.1	Forward: ACAGCCAGAAGAGATACGAA Reverse: ATTGCCATCTTTCCGGGTA	182
ACTC1	XM_027458711.2	Forward: ACCAACTGGGACGATATGGA Reverse: GCATAGAGGGACAGGACAGC	203

Abbreviation: DEGs, differentially expressed genes; qRT-PCR, quantitative real-time PCR.
